# EXIT Charts for Low-Density Algebra-Check Codes

**DOI:** 10.3390/e26121118

**Published:** 2024-12-20

**Authors:** Zuo Tang, Jing Lei, Ying Huang

**Affiliations:** College of Electronic Science and Technology, National University of Defense Technology, Changsha 410073, China; tz807016280@163.com (Z.T.); inform_huang@sina.com (Y.H.)

**Keywords:** EXIT, LDAC, LDPC, CND, algebraic codes, iterative decoder, channel coding

## Abstract

This paper focuses on the Low-Density Algebra-Check (LDAC) code, a novel low-rate channel code derived from the Low-Density Parity-Check (LDPC) code with expanded algebra-check constraints. A method for optimizing LDAC code design using Extrinsic Information Transfer (EXIT) charts is presented. Firstly, an iterative decoding model for LDAC is established according to its structure, and a method for plotting EXIT curves of the algebra-check node decoder is proposed. Then, the performance of two types of algebra-check nodes under different conditions is analyzed via EXIT curves. Finally, a low-rate LDAC code with enhanced coding gain is constructed, demonstrating the effectiveness of the proposed method.

## 1. Introduction

In modern communication systems, channel coding techniques play a crucial and fundamental role. Currently, research on channel coding primarily focuses on medium-to-high-rate codes to achieve high-speed and reliable transmission of big data. As society’s reliance on information and communication networks grows, the demand for communication in various scenarios rises. Low-rate channel coding technology is of great significance for improving communication capabilities and ensuring Minimum Essential Emergency Communication (MEEC) in extremely adverse channel conditions. For example, in Internet of Things (IoT) applications where sensor nodes transmit small amounts of data with high reliability requirements, or in emergency communication in remote areas with limited bandwidth and harsh environments. Obviously, a low-rate channel coding scheme is an indispensable part of the channel coding system.

However, research on low-rate channel coding techniques remains insufficient. Moreover, the performance of common channel coding schemes with excellent performance in medium-to-high rates, such as turbo code, Low-Density Parity-Check (LDPC) code, and polar code, significantly declines when constructing a low-rate channel code. The LDPC code is widely used in modern communication systems and exhibits excellent performance in medium-to-high-rate codes. However, the construction of low-rate LDPC codes is very difficult. The CCSDS [1] standard uses LDPC codes with a minimum rate of 1/2. The DVB-S2 standard [2,3] improves the coding performance by concatenating LDPC codes with BCH codes and applies LDPC codes with a minimum rate of approximately 1/4. Until now, there has been no uniform standard for channel coding with a rate less than 1/2.

Despite the challenges associated with low-rate LDPC codes, some attempts have been made to develop efficient low-rate channel coding [4,5,6,7,8,9]. However, these studies failed to break through the LDPC code structure and only achieved rate compatibility. Therefore, the construction of these codes is still under difficult conditions caused by reducing the rate of LDPC codes, often associated with issues such as complex construction and poor flexibility.

Generalized Low-Density Parity Check (GLDPC) codes [10,11] provide another perspective, showing that the check nodes of LDPC codes need not be limited to the Single Parity Check (SPC) and can be extended to other shortcodes with stronger error correction ability. Another code that extends the SPC node is the LDPC-Hadamard code [12], which stems from the parallel iterative decoding structure. Around the same period, the Turbo-Hadamard code [13,14] and the Zigzag-Hadamard code [15], both employing a similar structure, were also proposed, and all exhibit excellent performance at low rates. While the authors only considered approaching the Shannon limit, the length of designed codes was extremely long and difficult to implement in practice. In 2021, a team from Tsinghua University adopted the same structure as LDPC-Hadamard codes to propose LDBCH codes [16]. Subsequently, they further extended it and proposed Generalized Sparse (GS) Codes [17], which further enriched the types of subcodes.

Recently, a novel channel coding scheme named Low-Density Algebra-Check (LDAC) codes has been introduced, which is specifically designed to enhance the coding gain of low-rate codes [18]. In this paper, the Extrinsic Information Transfer (EXIT) chart [19,20,21] is used to optimize the design of LDAC codes. An iterative decoding model for LDAC codes is established, and the methods for plotting EXIT curves for two types of algebra-check nodes have been proposed. The performance of two types of algebra-check nodes of LDAC codes in different cases is discussed.

## 2. System Model

### 2.1. The Structure of LDAC Code

The LDAC code is derived from the LDPC code. To enhance the coding structure, LDAC codes incorporate complex algebraic checks to constrain the check nodes. Algebra-check constraints use algebraic codes as subcodes, which usually have significant algebraic structures, such as BCH codes [22,23,24], RS codes [25], and Hamming codes [26]. The nodes constrained by algebraic checks are named algebra-check nodes, and the constraint relationship of each algebra-check node can be represented by a set of algebraic equations or matrices. Based on whether all bits of the algebraic code take part in the decoding iteration, the algebra-check nodes are classified into two categories. These two types of algebra-check nodes are different in connection and will show different performance in decoding iteration.

**Figure 1:**
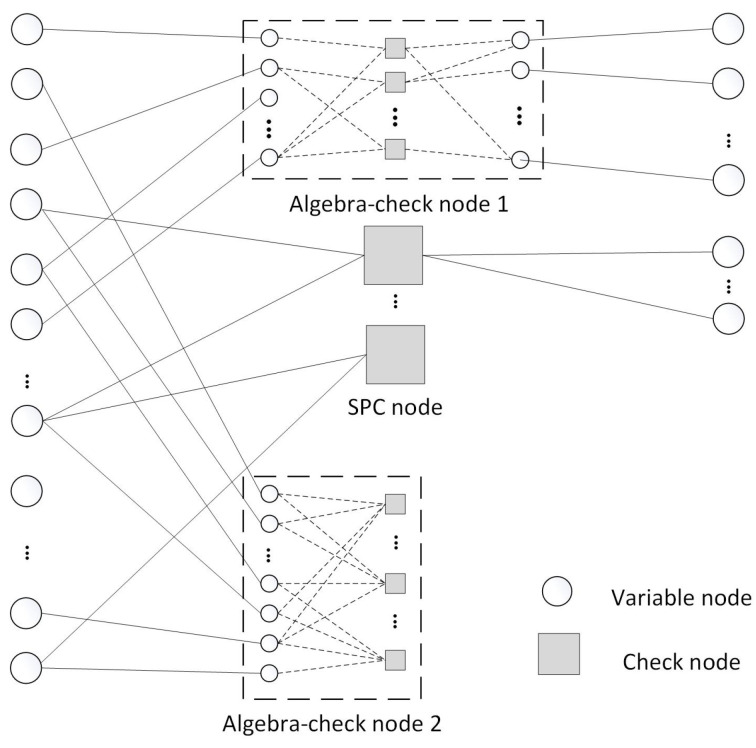
The Tanner graph of LDAC codes.

Figure 1 depicts the Tanner graph of LDAC codes. In this figure, a circle represents a variable node, a square represents a check node, and the Tanner graph of two types of algebra-check nodes is presented within the dotted box.

Each check node can be considered a subcode. For instance, an SPC node is related to an SPC code. In both LDAC and LDPC codes, all subcodes are connected in parallel based on the connection shown by the sparse check matrix. LDPC codes consist of SPC codes.

If an LDPC code has *M* SPC check nodes and its row degree is denoted as $\left(d_{1},d_{2},\ldots ,d_{M}\right)$, then the $i^{th}$ SPC check node corresponds to a $\left(d_{i},d_{i}-1\right)$ SPC subcode.

When the $i^{th}$ row is extended from the SPC check node to the first type of algebra-check node, the subcode related to it has $d_{i}-1$ information bits, and its check bits are more than 1. If the subcode length is $n_{i}$, this subcode can be represented as an ($n_{i}$, $d_{i}-1$) code. The specific structure of the first type of algebra-check node is displayed in algebra-check node 1 of Figure 1. The added check bits of the first type of algebra-check node are corresponded to the variable nodes with $d_{v}=1$, as shown in the right of the algebra-check node 1. In order to simplify the encoding and decoding operations of LDAC codes, when constructing the first type of algebra-check nodes, the first $d_{i}$ variable nodes still satisfy the SPC constraint.

When the $i^{th}$ row is extended from the SPC check node to the second type of algebra-check node, the length of the associated subcode is $d_{i}$. If the information bits have a length of $k_{i}$, the corresponding subcode can be represented as a ($d_{i}$, $k_{i}$) code. The specific structure is displayed in algebra-check node 2 of Figure 1.

Compared with LDPC codes, due to the enhanced error correction capabilities of the two types of algebra-check nodes, LDAC codes can construct low-rate to ultra-low-rate codes without introducing short cycles. The utilization of two types of algebra-check nodes provides more flexibility and reliability in LDAC code design, facilitating the construction of ultra-low-rate codes.

### 2.2. The Decoding Algorithm of LDAC Code

The decoding algorithm designed for LDAC codes must be suitable for SPC check nodes and two types of algebra-check nodes. Except for the check nodes, the general structure of the LDAC code closely resembles that of LDPC codes. Consequently, the Log-MAP-SPA decoding algorithm of LDAC codes is a combination of the Maximum a Posteriori Probability (MAP) decoding algorithm and the Log-Sum-Product Algorithm (Log-SPA). The key difference between Log-MAP-SPA and Log-SPA is in the update of check nodes.

Let ${\left\{L_{cv}\left(i,j\right)\right\}}_{i\in \left\{1,\ldots ,M\right\},j\in \left\{1,\ldots ,d_{i}\right\}}$ represent the message passing from check nodes to variable nodes, and ${\left\{L_{vc}\left(i,j\right)\right\}}_{i\in \left\{1,\ldots ,M\right\},j\in \left\{1,\ldots ,n_{i}\right\}}$ represent the message passing from variable nodes to check nodes. The codeword set of the subcode is denoted by C. At the beginning of the iteration, the message $L_{vc}\left(i,j\right)$ is from the extrinsic information received by the channel.

The iterative decoding message update of check nodes is

(1) $$L_{cv}\left(i,j\right)=\ln\frac{\sum_{t}^{\phi_{x_{k}=1}}\left(e^{0.5\sum_{j=1}^{N}\left(L_{vc}\left(i,j\right)\omega \left(c_{t,j}\right)\right)}\right)}{\sum_{t}^{\phi_{x_{k}=0}}\left(e^{0.5\sum_{j=1}^{N}\left(L_{vc}\left(i,j\right)\omega \left(c_{t,j}\right)\right)}\right)}-L_{vc}\left(i,j\right)$$

where $\phi_{x_{k}}=1/0$ denote the subsets of C with the $j^{th}$ is 1 or 0.

The iterative decoding message update of variable nodes can be written as

(2) $$L_{vc}\left(i,j\right)=\sum_{i^{\prime }\in VN\left(j\right)\setminus \left\{i\right\}}L_{cv}\left(i^{\prime },j\right)$$

where VN(j) represents the set of the check nodes positions connected to the $j^{th}$ variable node.

The range of *j* in $L_{cv}$ and $L_{vc}$ is different. The index *j* of message $L_{cv}\left(i,j\right)$ ranges from 1 to $d_{i}$, while the index *j* of message $L_{vc}\left(i,j\right)$ ranges from 1 to $n_{i}$. For the SPC check node, or the second type of algebra-check node, since $n_{i}=d_{i}$, all the variable nodes can be updated according to Formula (2). However, when it comes to the first type of algebra-check node, since $n_{i}>d_{i}$, it implies that the $n_{i}-d_{i}$ variable nodes cannot be updated. These $n_{i}-d_{i}$ variable nodes have $d_{v}=1$. The message $L_{vc}\left(i,j\right)$ with $j\in \{d_{i}+1,\ldots ,n_{i}\}$ (when $n_{i}>d_{i}$) is the extrinsic information from the channel and remains unchanged during the iterative decoding process.

### 2.3. Iterative Decoder

EXIT charts are utilized to analyze the input–output mutual information interactions between the Check Nodes Decoder (CND) and the Variable Nodes Decoder (VND), which is an effective method to predict the convergence of the iterative decoding process. Specifically, the CND updates the decoding message in accordance with Formula (1), while the VND updates the decoding message based on Formula (2).

**Figure 2:**
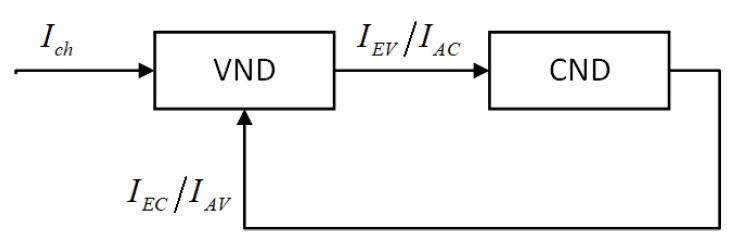
Block diagram of the iterative decoder for LDPC codes.

The LDAC codes employ an iterative decoding method, which is similar to LDPC codes. Therefore, the model of the LDAC iterative decoder can be derived from the model of the LDPC iterative decoder. The update message between the CND and VND is quantified by mutual information. As shown in Figure 2, the iterative decoding of LDPC codes is conceptualized as the reciprocal transmission of mutual information between the CND and the VND. Of which, $I_{ch}$ denotes the mutual information originating from the channel. The subscripts A/E represent input/output, while C/V represent check nodes/variable nodes, respectively. The relationships $I_{EV}=I_{AC}$ and $I_{EC}=I_{AV}$ are maintained throughout this process.

For the VND of LDPC codes, $I_{EV}$ is a function of degree $d_{v}$, input mutual information $I_{AV}$, and channel mutual information $I_{ch}$.

(3) $$I_{EV}=f\left(I_{AV},I_{ch},d_{v}\right)$$

For the CND of LDPC codes, $I_{EC}$ is a function of degree $d_{c}$ and input mutual information $I_{EV}$.

(4) $$I_{EC}=g\left(I_{AC},d_{c}\right)$$

However, a CND with an algebra-check extension cannot be described by Function (4). The output $I_{EC}$ is a function of input mutual information $I_{EV}$, channel mutual information $I_{ch}$, and degree $d_{c}$.

(5) $$I_{EC}=g\left(I_{AC},I_{ch},d_{c}\right)$$

**Figure 3:**
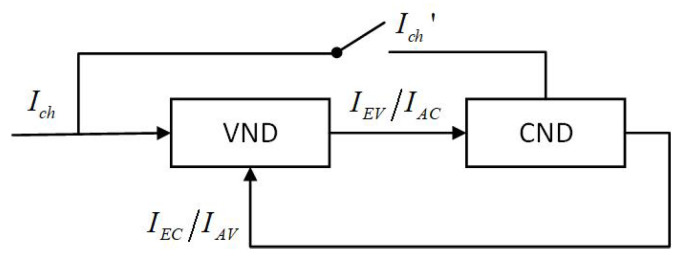
Block diagram of the iterative decoder for LDAC codes.

Depending on the selection of the check nodes, the input channel mutual information $I_{ch}$ may be zero. The block diagram of the iterative decoder for LDAC codes is presented in Figure 3, and the meaning of symbols in the figure refers to Figure 2.

### 2.4. Calculation of Mutual Information

The mutual information can be calculated according to the log-likelihood ratio (LLR) of the transmitted bits. Consider the AWGN channel with BPSK modulation. Assume that the transmitted bit is *x*, and the information received from the channel is *y*. The calculation of LLR is presented as follows:

(6) $$l=\log\frac{p\left(\left.x=1\right|y\right)}{p\left(\left.x=0\right|y\right)}=\frac{2}{\sigma_{n}^{2}}y$$

$\sigma_{n}^{2}$ is noise variance. Then, the conditional probability density function of the LLR is

(7) $$f_{L}\left(\left.l\right|X=x\right)$$

Define mutual information $I_{L}$:

(8) $$I_{L}=I\left(X;L\right)$$

Through derivation, we can obtain

(9) $$I_{L}=\frac{1}{2}\sum_{X}\int_{-\infty }^{+\infty }f_{L}\left(l\left|X=x\right.\right)\cdot \log_{2}\frac{2f_{L}\left(l\left|X=x\right.\right)}{f_{L}\left(l\left|X=0\right.\right)+f_{L}\left(l\left|X=1\right.\right)}dl$$

Among which, *X* is the transmitted bit, and *L* is the LLR of extrinsic information.

If *L* satisfies symmetry and consistency:

(10) $$f_{L}\left(l\left|X=1\right.\right)=f_{L}\left(-l\left|X=0\right.\right)$$

(11) $$f_{L}\left(-l\left|X=x\right.\right)=e^{-l}f_{L}\left(l\left|X=x\right.\right)$$

and the conditional probability density function of *L* follows a Gaussian distribution. The corresponding distribution function is

(12) fLlX=1=12πσe−l−σ2σ22222σ2

(13) fLlX=0=12πσe−l+σ2σ22222σ2

Substitute (12) and (13) into (9) to obtain

(14) IL=1−12πσ∫−∞∞e−l−σ2σ22222σ2log21+e−ldl

Definition function:

(15) Jσ=1−12πσ∫−∞∞e−y−σ2σ22222σ2log21+e−ydy

Obviously, $\sigma =J^{-1}\left(I\right)$. The functions *J* and $J^{1}$ can be segmented for several approximate and simplified calculations as cited in [20].

## 3. EXIT Curves of LDAC Codes

In LDAC codes, the EXIT curves of VND are identical to those of the LDPC code. However, the CND of LDAC codes contains three possible cases. In this section, the EXIT curves for both the VND and the three different types of CND are introduced, respectively.

### 3.1. VND

Similar to LDPC codes, the VND of LDAC codes can be regarded as a repetition (REP) code decoder. We define the normalized signal-to-noise ratio (SNR) as $E_{b}/N_{0}=1/\left(2R\sigma_{n}^{2}\right)$, where *R* is the rate of the LDAC code. The mutual information between the input and output of the VND at the same degree $d_{v}$ can be calculated by Formula (9).

(16) $$I_{EV}=J\left(\sqrt{\left(d_{v}-1\right){\left[J^{-1}\left(I_{AV}\right)\right]}^{2}+{\left[J^{-1}\left(I_{ch}\right)\right]}^{2}}\right)$$

(17) σch2=J−1Ich2=8R·EbEbN0N0

**Figure 4:**
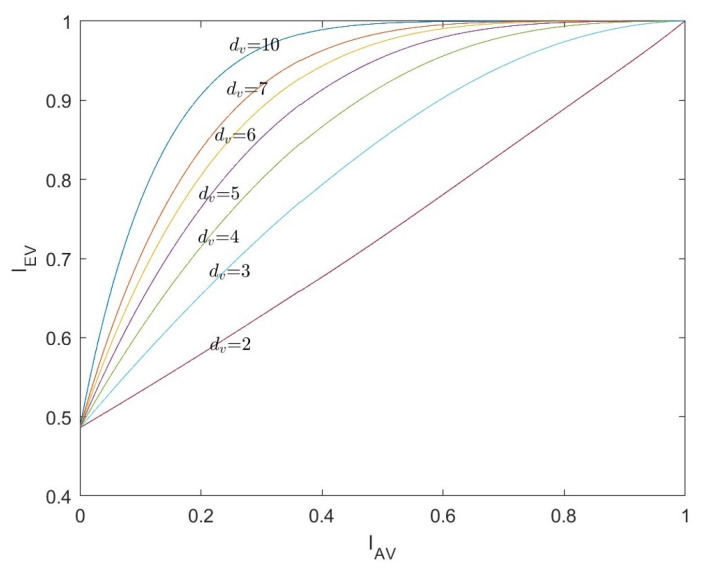
The EXIT curves of VND with EbEbN0N0=2dB,R=1/2.

*R* is the rate of the LDAC code. The EXIT curves of VND with different degrees $d_{v}$ are shown in Figure 4.

### 3.2. CND

The check nodes of LDAC codes include the SPC node, the first type of algebra-check node, and the second type of algebra-check node.

The SPC can be considered as the dual of repetitive codes, and the EXIT curve of the SPC node is only related to the degree $d_{c}$ of the check node. The relationship between the input and output mutual information of the VND with only one degree $d_{c}$ is as follows:

(18) $$I_{EC}^{SPC}=1-I^{REP}=1-J\left(\sqrt{\left(d_{c}-1\right){\left[J^{-1}\left(1-I_{AV}\right)\right]}^{2}}\right)$$

**Figure 5:**
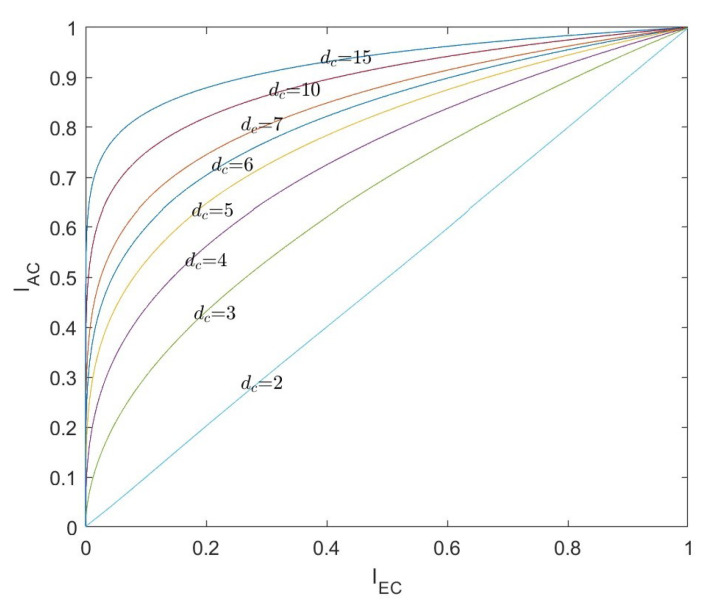
The EXIT curves of the SPC nodes decoder.

The EXIT curves of the SPC nodes decoder with different degrees of $d_{c}$ are shown in Figure 5.

In LDAC codes, the two types of algebra-check nodes are distinct from the SPC nodes. The decoding output of an algebra-check node decoder is related to the specific algebra-check constraint. The calculation is more complex than the SPC decoder, and it is difficult to obtain the specific expression of each constraint. Therefore, for the algebra-check nodes decoder, the Monte Carlo method is employed to plot the EXIT curves.

For a given input mutual information value $I_{AC}$, according to the experimental results in Reference [20], when the number of experiments is large enough, the conditional probability functions $P\left(L_{A}|X=0\right)$ and $P\left(L_{A}|X=1\right)$ can be considered to follow a Gaussian distribution. Therefore, under the circumstances of fulfilling the symmetry and consistency conditions, when the variance is $\sigma_{A}^{2}$, the mean can be calculated as $\sigma_{A}^{2}/2$. The expression of the conditional probability functions is as follows:

(19) PLA=lX=x=12πσAe−l−σA2σA222ωx22σA2

Substitute Formula (19) into the mutual information calculation Formula (9) to obtain

(20) $$I_{AC}=J\left(\sigma_{A}\right)$$

When the input mutual information value $I_{AC}$ is known, obtain

(21) $$\sigma_{A}=J^{-1}\left(I_{AC}\right)$$

When the output LLR sequence $L_{E}$ is known and the sequence size is sufficiently large, the empirical distribution of the output can be obtained via the Monte Carlo method, which is regarded as an approximation of the probability distribution. As a result, the conditional probability density function $P\left(L_{E}=l|X=x\right)$ can be obtained, and the output mutual information $I_{EC}$ can be calculated according to Formula (9). In addition, according to Formula (17), the variance of channel information ${\sigma_{ch}}^{2}$ can be obtained. Two types of algebra-check nodes of LDAC codes are analyzed below.

According to the decoding algorithm of LDAC codes [18], if the $i^{th}$ check node is the first type of algebra-check node, only the first $d_{i}$ variable nodes connected to it participate in the decoding iteration. The latter $n_{i}-d_{i}$ variable nodes only provide the extrinsic information from the channel but remain unchanged during the iterative decoding process. Therefore, for the first type of algebra-check node, the output mutual information $I_{EC}$ of the CND is a function of the input mutual information $I_{AC}$ and the extrinsic mutual information from the channel $I_{ch}$. And the vector input of the CND is the vector $\left(L_{A,1},L_{A,2},\ldots ,L_{A,d_{c}},L_{ch,1},L_{ch,2},\ldots ,L_{ch,n-d_{c}}\right)$. If the $i^{th}$ check node is the second type of algebra-check node, the output mutual information $I_{EC}$ of the CND is only related to the input mutual information $I_{AC}$, and every variable node connected to the check node updates during the iterative decoding. Therefore, for the second type of algebra-check node, the vector input of the CND is the vector $\left(L_{A,1},L_{A,2},\ldots ,L_{A,d_{c}}\right)$.

For the first type of algebra-check node, the process for plotting the EXIT curves of the CND by the Monte Carlo method is as follows:

1. Set the value of input mutual information $I_{AC}$ ranging from 0 to 1, and set the target rate *R* and $E_{b}/N_{0}$;
2. Calculate $\sigma_{ch}$ according to *R* and $E_{b}/N_{0}$, and randomly generate the channel information $L_{ch}$;
3. Calculate $\sigma_{A}$ according to $I_{AC}$ and randomly generate the input $L_{A}$ of the CND.
4. Input $L_{ch}$ and $L_{A}$ into the CND and calculate the output message $L_{EC}$ according to Formula (1);
5. Accumulate sufficient output messages $L_{E}$ for empirical distribution. Consider it as a probability distribution to obtain the conditional probability density function of $L_{E}$ and calculate the output mutual information $I_{EC}$;
6. Plot the EXIT curves of the first type of algebra-check node decoder, according to the input mutual information $I_{AC}$ and output mutual information $I_{EC}$.

For the second type of algebra-check node, the process for plotting the EXIT curves of the CND by the Monte Carlo method is as follows:

1. Set the value of input mutual information $I_{AC}$ ranging from 0 to 1;
2. Calculate $\sigma_{A}$ according to $I_{AC}$ and randomly generate the input $L_{A}$ of the CND;
3. Input $L_{A}$ into the CND and calculate the output message $L_{EC}$ according to Formula (1);
4. Accumulate sufficient output messages $L_{E}$ for empirical distribution. Consider it as a probability distribution to obtain the conditional probability density function of $L_{E}$ and calculate the output mutual information $I_{EC}$;
5. Plot the EXIT curves of the second type of algebra-check node decoder, according to the input mutual information $I_{AC}$ and output mutual information $I_{EC}$.

**Figure 6:**
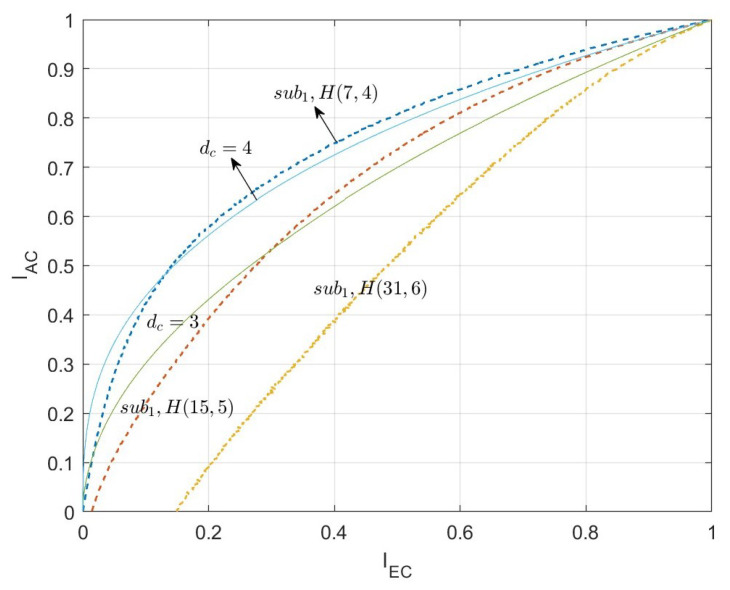
The EXIT curves of the first type of algebra-check node decoder.

**Figure 7:**
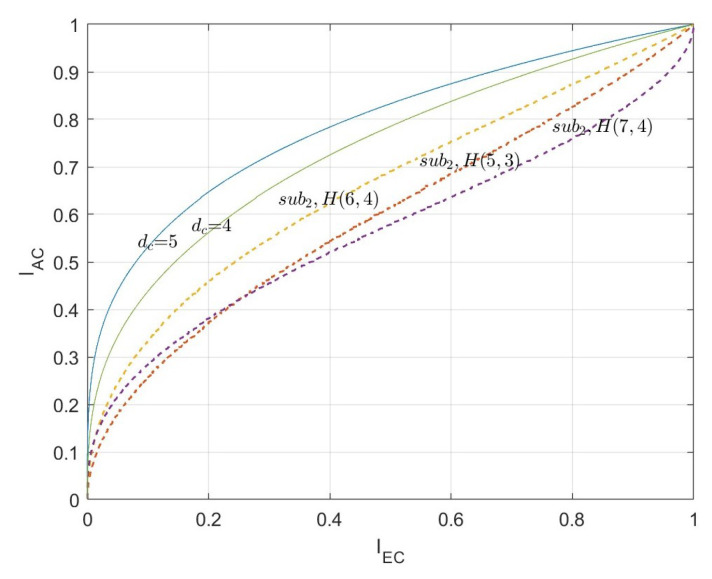
The EXIT curves of the second type of algebra-check node decoder.

According to the above steps, for the two types of algebra-check nodes, the EXIT curves of the CND are simulated respectively, as shown in Figure 6 and Figure 7. The curves indicated by $d_{c}=3$, $d_{c}=4$, and $d_{c}=5$ are the EXIT curves of the SPC node decoder with the corresponding degree. The curves labeled by $sub_{1}H\left(31,6\right)$, $sub_{1}H\left(15,5\right)$, and $sub_{1}H\left(7,4\right)$ are the EXIT curves of the first type of algebra-check node with the modified BCH codes. Specifically, the code lengths are 31, 15, and 7, respectively. Associated with the information bit length are 6, 5, and 4, respectively. Corresponding to the row degree, $d_{c}=7$, $d_{c}=6$, and $d_{c}=5$, respectively. The curves labeled by $sub_{2}H\left(7,4\right)$, $sub_{2}H\left(6,4\right)$, and $sub_{2}H\left(5,3\right)$ are the EXIT curves of the first type of algebra-check node with the Hamming codes. Specifically, the code lengths are 7, 6, and 5, respectively. Associated with the information bit lengths are 4, 4, and 3, respectively. Corresponding to the row degree, $d_{c}=7$, $d_{c}=6$, and $d_{c}=5$, respectively.

In Figure 6 and Figure 7, the shape of the EXIT curves of the algebra-check nodes significantly differs from that of the SPC nodes. Especially for the first type of algebra-check nodes, the EXIT curves of the CND do not pass through the origin but intersect with the X-axis. This is because there is mutual information from the channel inputted into the CND.

## 4. Simulation Results and Analysis

### 4.1. The Analysis of the VNDs and SPC Node

**Figure 8:**
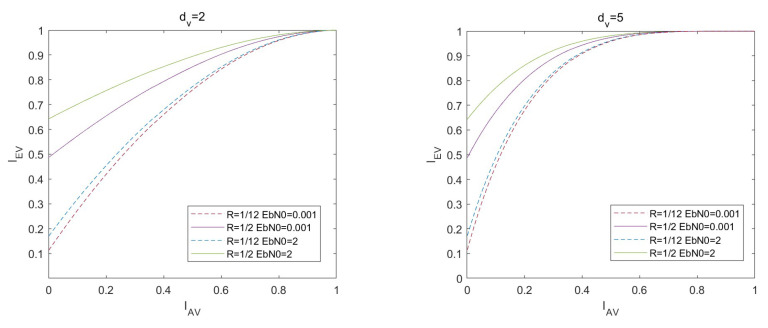
EXIT curves of VND under different conditions.

Figure 8 shows the VND EXIT curves with $d_{v}=2$ and $d_{v}=5$ given different code rates *R* and $E_{b}/N_{0}$. Comparing the EXIT curves about the rate of 1/2 and 1/12, it is observed that the intersection of the EXIT curves with the Y-axis approaches the origin as the code rate decreases. Moreover, when the bit rate is 1/12, increasing the $E_{b}/N_{0}$ from 0.001 dB to 2 dB, the EXIT curves are significantly smaller than the EXIT curves with the rate of 1/2. It is observed that the impact of varying $E_{b}/N_{0}$ on the VND EXIT curves diminishes as the code rate decreases. This indicates that reducing the code rate makes it increasingly challenging to enhance the output performance of the variable node decoder by merely increasing $E_{b}/N_{0}$.

**Figure 9:**
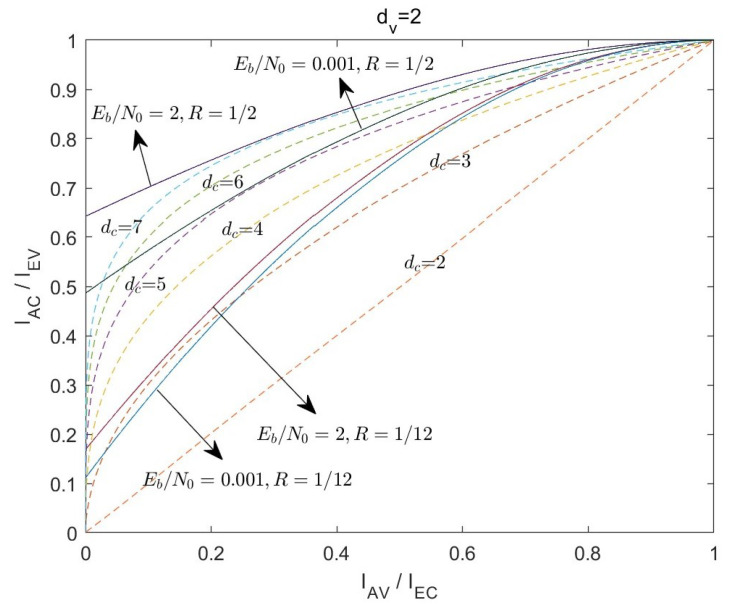
EXIT curves of VND and SPC node decoder at different rates.

To achieve the iterative decoding, the VND curve in the EXIT chart must surpass the CND curve, ensuring that the two curves remain non-intersecting and establishing a decoding channel. Figure 9 depicts the VND EXIT curves for $d_{v}=2$ at different bit rates. Additionally, the figure presents CND EXIT curves with different degrees $d_{c}$. It can be directly observed that when R=1/2 and $E_{b}/N_{0}=2\mathrm{dB}$, the CND EXIT curves with $d_{c}\leq 7$ are below the VND EXIT curve with $d_{v}=2$. When R=1/2 and $E_{b}/N_{0}=0.001\;\mathrm{dB}$, the CND EXIT curves with $d_{c}\leq 5$ are below the VND EXIT curve with $d_{v}=2$. When R=1/12 and $E_{b}/N_{0}=2\;\mathrm{dB}$, only the CND EXIT curves with $d_{c}=3$ and $d_{c}=2$ are below the VND EXIT curve with $d_{v}=2$. When R=1/12 and $E_{b}/N_{0}=0.001\;\mathrm{dB}$, only the CND EXIT curve with $d_{c}=2$ is below the VND EXIT curve with $d_{v}=2$.

The analysis reveals that when the code rate reduces, the variable node degree $d_{v}$ necessary for achieving iterative decoding increases, while the check node degree $d_{c}$ decreases. This implies that the matrix of the low-rate codes must be structured with the higher column degrees and the lower row degrees. However, it is impossible to decrease the row degree $d_{c}$ and increase the column degree $d_{v}$ at the same time, because the sum of row degrees is always equal to the sum of column degrees. If the row degree $d_{c}$ decreases, the CNDs will become insufficiently connected, leading to poor performance during decoding. Conversely, if the column degree $d_{v}$ decreases, the number of short cycles tends to increase, which also adversely affects the decoding performance.

The construction of low-rate LDPC codes presents considerable challenges, and the introduction of algebra-check nodes provides a new ideal for constructing low-rate codes. By modifying the structure of check nodes, its decoding capabilities can be enhanced. The change in the shape of CND EXIT curves can optimize iterative decoding performance without modifying $d_{v}$ and $d_{c}$. This offers another option for constructing a low-rate code.

### 4.2. The Comparison of Algebra-Check Nodes

Initially, to evaluate and contrast the performance of the two types of algebra-check nodes, the impact on the rate of LDAC codes following the substitution of these check nodes is analyzed. The detailed derivation process is as follows.

Suppose the increase of the code length due to the first type of algebra-check nodes is *b*. The reduction of the information bits due to the second type of algebra-check nodes is *a*. The LDAC code’s basis matrix is $H_{0}$, with a code length of $N_{H_{0}}$ and a rate of $R_{0}$. The final rate of the LDAC code is *R*.

(22) $$R=\frac{R_{0}N_{H_{0}}-a}{N_{H_{0}}+b}$$

If a=Rb, then

(23) $$R=\frac{R_{0}N_{H_{0}}-Rb}{N_{H_{0}}+b}$$

When R>a/b, there is

(24) $$\frac{R_{0}N_{H_{0}}-a}{N_{H_{0}}+b}>\frac{R_{0}N_{H_{0}}-Rb}{N_{H_{0}}+b}$$

When R<a/b, there is

(25) $$\frac{R_{0}N_{H_{0}}-a}{N_{H_{0}}+b}<\frac{R_{0}N_{H_{0}}-Rb}{N_{H_{0}}+b}$$

Thus, the following relationships can be obtained:

- If R>a/b, substituting the first type of algebra-check nodes leads to a more significant rate loss compared to replacing the second type.
- When R<a/b, replacing the first type of algebra-check nodes incurs a relatively smaller rate loss than substituting the second type.
- In the case of R=a/b, the influence of both types of algebra-check nodes on the rate of the constructed LDAC code is comparable.

For a specific analysis, the comparison of algebra-check nodes requires the combination with parameters *R*, $E_{b}/N_{0}$, and the ratio a/b. Here, the cases with code rates R=1/12 and R=1/4 are discussed as examples.

(1) The constructed LDAC code with the target rate R=1/12.

**Figure 10:**
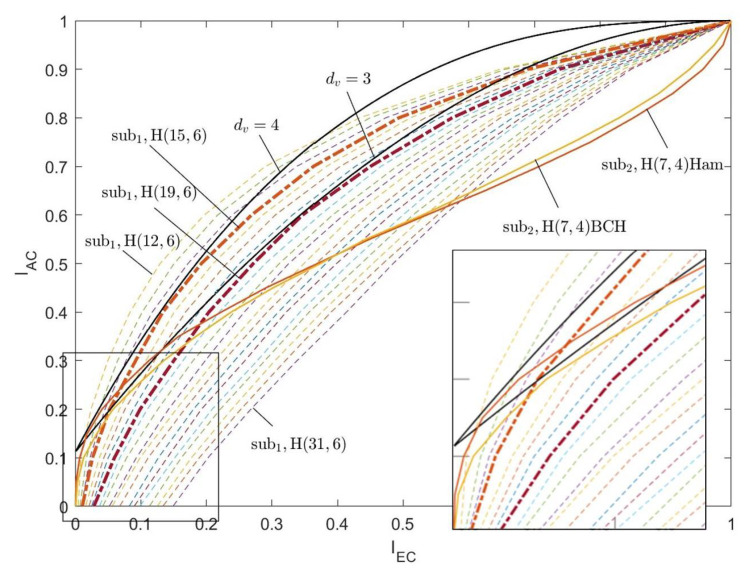
The EXIT curves of two types of algebra-check nodes with the target rate R=1/12.

Figure 10 shows the EXIT curves of different algebra-check nodes with the target rate R=1/12 and $E_{b}/N_{0}=0.001\;\mathrm{dB}$. The symbols ${\mathrm{sub}}_{1}$ and ${\mathrm{sub}}_{2}$ denote the first and the second type of algebra-check nodes, respectively. The EXIT curves of the first type of algebra-check nodes are all restricted by the modified BCH codes, while the EXIT curves of the first type of algebra-check nodes are constrained by the modified BCH codes and Hamming codes.

When the rate of the basis matrix is $R_{H_{0}}=1/2$, the degrees of the variable nodes are mainly 3 and 4. Thus, the EXIT curve of variable node decoders with $d_{v}=3$ and $d_{v}=4$ is presented in the figure as a reference.

For the second type of algebra-check nodes, the selected algebra-subcodes are the (7,4) Hamming code and the (7,4) modified BCH system code. The EXIT curve of the (7,4) Hamming code is always below the VND EXIT curve of the $d_{v}=4$, yet intersects with the VND EXIT curve of $d_{v}=3$. While the EXIT curve of the (7,4) modified BCH code below both the VND EXIT curves of $d_{v}=3$ and $d_{v}=4$. The simulation results indicate that the second type of algebra-check node exhibits superior iterative convergence performance compared to the SPC node, and the (7,4) modified BCH code outperforms the Hamming code.

For the first type of algebra-check node, When the subcode of the information bit $K^{\mathrm{sub}1}=6$ and the length of code $N^{\mathrm{sub}1}<30$, the first type of algebra-check node has a rate advantage compared to the second type of algebra-check node with a (7,4) code. The dotted lines in Figure 10 display the EXIT curves for the first type of algebra-check node with $K^{\mathrm{sub}1}=6$. The subcode lengths $N^{\mathrm{sub}1}$ range from 12 to 31, increasing from left to right. The curves closest to $d_{v}=3$ and $d_{v}=4$ are the curves of the (15,6) code and the (19,6) code, with code rates of 0.4 and 0.316, corresponding to a/b=1/4 and a/b=1/6, respectively.

In summary, if the rate of LDAC code is 1/12, for a check node with row degree $d_{c}=7$, the first type of algebra-check nodes with a code length greater than 15 and the second type of algebra-check nodes with the (7,4) code can be considered as candidates.

(2) The constructed LDAC code with the target rate R=1/4.

**Figure 11:**
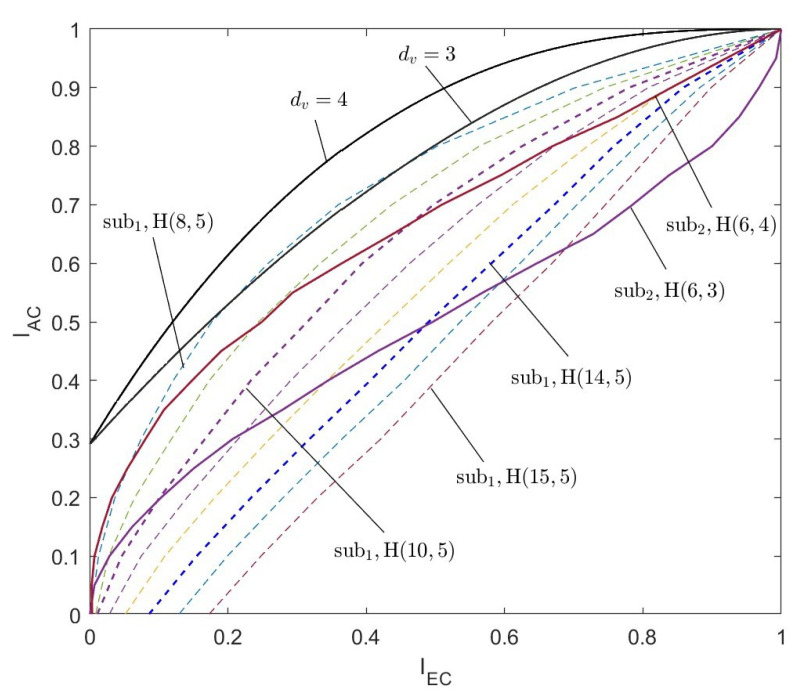
The EXIT curves of two types of algebra-check nodes with the target rate R=1/4.

The second type of algebra-check nodes that participate in the comparison is the (6,4) code and (6,3) code, corresponding to a=1 and a=2, respectively. The two EXIT curves in Figure 11 are located below the EXIT curve of $d_{v}=3$ and are significantly lower than the VND EXIT curve of $d_{v}=4$.

The first type of algebra-check node with $K^{\mathrm{sub}1}=5$ and $N^{\mathrm{sub}1}<14$ has an advantage over the second type of algebra-check node with the (6,3) code. And the first type of algebra-check node with $K^{\mathrm{sub}1}=5$ and $N^{\mathrm{sub}1}<10$ has an advantage over the second type of algebra-check node with the (6,4) code.

The dotted lines in Figure 11 display the EXIT curves for the first type of algebra-check node with $K^{\mathrm{sub}1}=5$. The subcode lengths $N^{\mathrm{sub}1}$ increase from left to right, starting at 8 and ending at 15. Except the curves of (8,5) code intersect with the VND EXIT curve of $d_{v}=3$, the other curves do not intersect with the VND EXIT curves of $d_{v}>2$.

In summary, for the LDAC code construction of the target rate R=1/4, both types of algebra-check nodes show great performance.

### 4.3. Examples of Optimized Constructions of LDAC Codes

In response to the severe channel conditions with extremely low SNR, we have constructed LDAC codes with a code rate of R=1/12. The selection of check nodes is detailed in Table 1.

**Figure 12:**
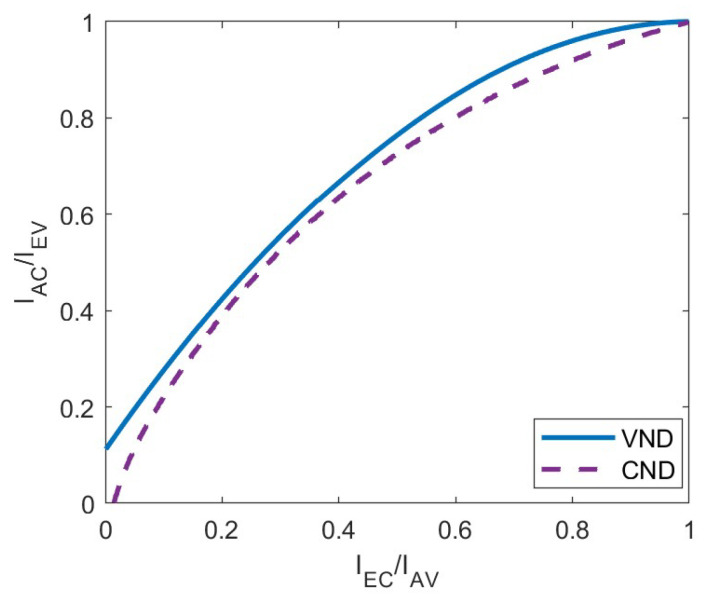
EXIT chart of LDAC code with rate R=1/12.

The EXIT chart for the LDAC code at R=1/12 and $E_{b}/N_{0}=0.001$ dB is presented in Figure 12.

**Figure 13:**
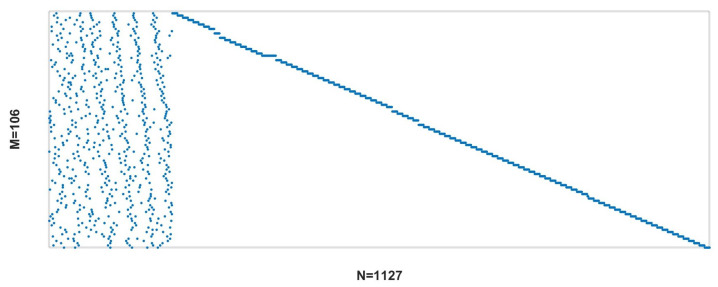
Decoding matrix of LDAC code with code rate R=0.0834.

The LDAC decoding matrix H at R=1/12 is shown in Figure 13.

**Figure 14:**
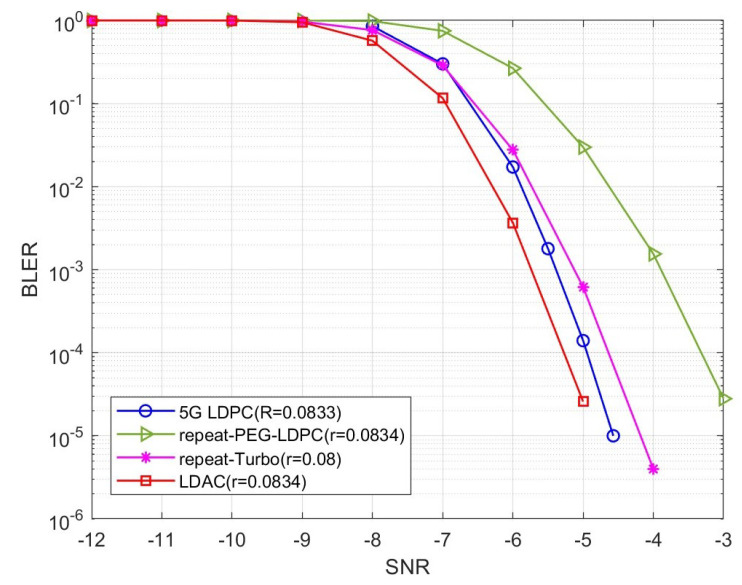
The BLER of LDAC code with R=0.0834.

Figure 14 shows the block error rate (BLER) of the LDAC code constructed based on the above parameters in the AWGN channel, comparing it with the 1/2-LDPC repetition code, 1/3-Turbo repetition code, and the 5G LDPC code [27]. The simulation results show that under QPSK modulation, the BLER of the LDAC code with R=0.0834 is significantly lower than the 1/2-LDPC repetition code and outperforms the 5G LDPC code and 1/3-Turbo repetition code, demonstrating superior performance.

## 5. Conclusions

In this paper, the optimization design of LDAC codes based on the EXIT chart is studied. An iterative decoder model for LDAC codes is proposed, along with calculation methods for the input–output mutual information curves of two types of algebra-check node decoders. The paper simulates various EXIT curves of VNDs and CNDs with different conditions, investigates the limitations of low-rate LDPC codes, and analyzes the performance of the two algebra-check nodes. Finally, based on the EXIT chart, the optimitive constructed LDAC code based on the EXIT chart. The simulation results show that the optimized LDAC code greatly reduces the BLER compared with the 1/2 LDPC repetition code and has enhanced coding gain compared with 5G LDPC code and 1/3-Turbo repetition code.

## Figures and Tables

**Table 1:** Selection of check nodes.

Degree $d_{c}$	The Type of Check Node	Subcode Length $n_{i}$	Information Bit Length $k_{i}$
5	sub1	7	4
6	sub1	15	5
7	sub1	31	6
7	sub2	7	4

## Data Availability

The data presented in this study are available on request from the corresponding author.

## Abbreviations

The following abbreviations are used in this manuscript:

EXIT	Extrinsic Information Transfer
LDPC	Low-Density Parity-Check
LDAC	Low-Density Algebra-Check
GLDPC	Generalized Low-Density Parity Check
SPC	Single Parity Check
CND	Check Nodes Decoder
VND	Variable Nodes Decoder
MEEC	Minimum Essential Emergency Communication
GS	Generalized Sparse
SNR	signal-to-noise ratio
BLER	block error rate
